# A Fully Automated Deep Learning Pipeline for Anatomical Landmark Localization on Three-Dimensional Pelvic Surface Scans

**DOI:** 10.3390/s26061760

**Published:** 2026-03-10

**Authors:** Woosu Choi, Jun-Su Jang

**Affiliations:** Korea Institute of Oriental Medicine, 1672 Yuseong-daero, Yuseong-gu, Daejeon 34054, Republic of Korea; harrius@kiom.re.kr

**Keywords:** deep learning, point cloud, pelvic ROI extraction, 3D alignment, landmark localization

## Abstract

Accurate identification of anatomical landmarks on three-dimensional (3D) pelvic surface scans is essential for musculoskeletal assessment, yet manual procedures remain limited by operator dependence and soft tissue variability. This study presents a fully automated deep learning pipeline for localizing anatomical landmarks on the posterior pelvic region from raw 3D point cloud data. The pipeline integrates three modules: PelvicROINet for extracting the region of interest, PelvicAlignNet for rotation correction to standardize posture, and PelvicLandmarkNet for localizing six anatomical landmarks including the bilateral posterior superior iliac spines, bilateral iliac crests, L1, and L4. The models were trained independently with task-specific annotations and combined sequentially during inference. Under a subject-level split evaluation setting, the fully integrated system achieved a median error of 11.25 mm, demonstrating consistent localization performance across unseen subjects. Compared with manual landmark marking, the automated measurements showed improved within-visit repeatability, with reduced variability and higher intraclass correlation coefficients. The entire inference process required approximately three seconds per scan, supporting near real-time clinical applicability. These results indicate that the proposed modular framework enhances numerical consistency and robustness in surface-based pelvic landmark assessment and provides a scalable foundation for AI-assisted musculoskeletal evaluation and longitudinal monitoring.

## 1. Introduction

Interest in quantitatively assessing human alignment and posture is steadily increasing in various clinical and biomechanical research fields [1,2]. In particular, the pelvis, as the connection point between the spine and the lower extremities, plays a key role in human alignment. Pelvic asymmetry and positional changes have been reported to be closely associated with back pain, gait abnormalities, and postural imbalances [3,4]. Against this background, there has been an increasing need for objective and non-invasive methods to assess pelvic shape and alignment.

Three-dimensional (3D) body surface scanning technology enables direct acquisition of external body shape [5] and is gaining attention as a suitable alternative for morphological analysis and evaluation of alignment of surface-based anatomical structures such as the pelvis, by offering non-invasive and accurate surface data for applications including posture evaluation [6], musculoskeletal diagnosis [7,8], and rehabilitation monitoring [9]. Conventional computed tomography (CT) or magnetic resonance imaging (MRI) provides high-resolution visualization of internal anatomical structures. However, limitations such as high cost, long acquisition time, and limited accessibility make repeated measurements difficult and restrict their use for evaluating immediate postural changes in clinical settings. In contrast, 3D body surface scanning allows repeated measurements through relatively simple procedures and offers advantages in terms of cost-effectiveness and accessibility, making it well suited for tracking postural or morphological changes before and after treatment [10,11].

Several technical limitations remain in the automatic interpretation of data acquired through 3D body surface scanning and in the reliable extraction of clinically meaningful information. Although most clinical studies acquire 3D body scan data in accordance with standardized operating procedures (SOP), automated interpretation of raw surface point clouds remains challenging due to measurement noise introduced during scanning, their high dimensionality and substantial inter-individual variability [12,13]. Classical image preprocessing methods, such as thresholding, region of interest (ROI) selection, and surface smoothing, have limitations in reliably distinguishing actual body surface changes from measurement noise [14]. As a result, clinically significant surface variations may be unnecessarily removed during preprocessing [15]. Beyond noise-related issues, substantial variations in body surface geometry are inevitable due to inter-subject differences in body shape. Moreover, repeated measurements can vary due to slight changes in posture or unintentional movements during scanning. The Iterative Closest Point (ICP) algorithm [16] has been widely used to align and normalize body surface data by correcting such variations. However, ICP is highly dependent on the initial alignment and frequently converges to local minima when large rotational differences are present or when parts of the surface data are missing [17]. These limitations can reduce reliability in practical clinical settings.

To establish clearer reference standards for clinical analysis, anatomical landmarks on the subject’s skin are sometimes manually marked using pen markers or by attaching physical markers. The resulting marker positions can be used as reference points for algorithm-based analysis and the extraction of clinically significant features. However, the process of manually marking anatomical locations on the body surface is highly dependent on the level of expertise of the examiner, leading to substantial variability both between examiners and across repeated measurements [18]. Furthermore, additional errors may be introduced during the digitization of marker locations after scanning [19]. Consequently, these traditional approaches remain inherently limited and are insufficient as a fundamental solution for fully automated 3D body surface analysis.

Recent advances in computing hardware and artificial intelligence (AI) algorithms have driven the adoption of deep learning techniques across a wide range of research fields, including medical imaging and biomechanical analysis. In particular, the emergence of 3D point cloud processing models such as PointNet [20] and PointNet++ [21] has enabled direct feature learning from raw 3D point sets without requiring complex voxelization procedures. This approach has improved computational efficiency while reducing memory requirements, allowing these models to be applied to real-time analysis and diagnostic tasks. Subsequently, numerous point-based deep learning models, including variants of PointNet/PointNet++ and other alternative architectures, have demonstrated strong performance in object classification [22], segmentation [23], and feature detection tasks [24].

However, extracting clinically significant anatomical features from 3D body surface scans remains a challenge. This difficulty arises because the surface of the human body is covered by soft tissue and internal skeletal landmarks are not directly represented on the external surface. Even for the same anatomical location, its position on the body surface can vary substantially depending on posture [25]. Movement of the subject during scanning can also cause the locations defined in a specific posture to appear in different surfaces [6]. In routine surface scanning workflows, complementary thermal or physical information that could provide additional physiological or biomechanical context is typically unavailable. Consequently, accurate pelvic landmark localization must rely primarily on surface-geometric cues alone, further increasing the difficulty of reliable detection. Although multimodal approaches integrating anatomical and thermal data have been proposed [26], they generally require additional hardware and complex acquisition procedures, limiting their practicality in routine clinical settings. Under these constraints, a single model trained to detect local features from dense 3D point clouds is prone to large errors when newly acquired data differ from the anatomical shapes or imaging conditions present in the training data. This challenge is further compounded by the limited availability of accurately labeled datasets during model training. Although precise labels are essential for supervised learning, annotating 3D scan data in clinical settings is time consuming and requires considerable manual effort [27]. Such processes are also inevitably affected by multiple sources of error, including differences in operator experience, subtle movements of subjects, and human error. To address these challenges and achieve robust generalization in diverse body types, postures, and imaging conditions, a modular architecture that combines multiple specialized models with distinct functions is required. Each model is trained independently to address specific sources of variability and later integrated to enhance the overall reliability of the system.

In this study, we propose a modular analysis framework for 3D human pelvic body surface analysis that sequentially performs body surface segmentation, alignment, and anatomical landmark localization. Three models are designed and optimized for each processing stage and their effectiveness is validated through quantitative and qualitative evaluations at the individual module level, as well as at the integrated pipeline level.

## 2. Materials and Methods

### 2.1. Dataset

**Figure 1:**
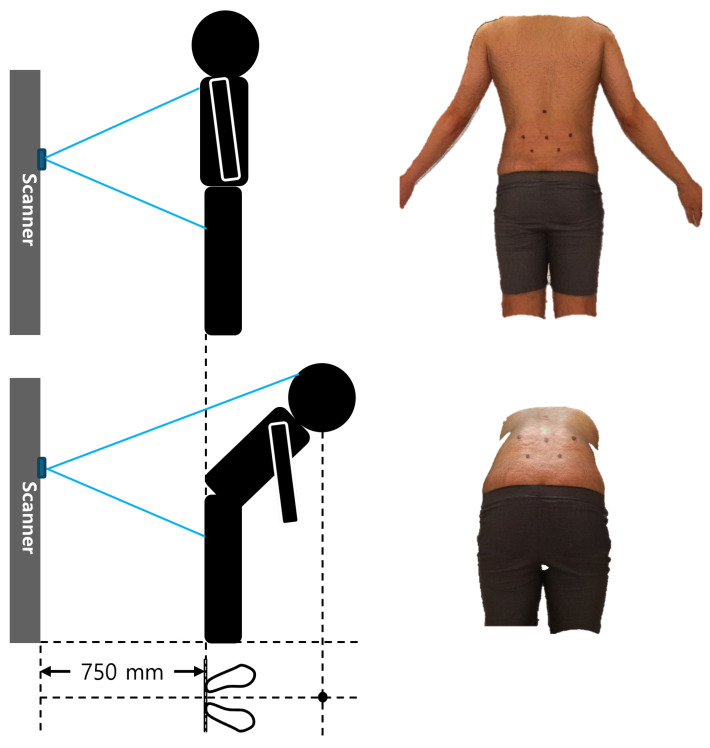
Measurement setup and postures for 3D surface scanning. Schematic diagrams (**left**) illustrate the scanner configuration and participant positions for postures P1 (upper) and P2 (lower), while the corresponding 3D surface images (**right**) show the actual scanned data.

The data were obtained from a single three-dimensional surface dataset covering both the back and pelvis regions using a structured-light 3D scanner (model: iBalance, TeamElysium, Seoul, Republic of Korea) equipped with a Kinect Azure sensor (model: Azure Kinect, Microsoft, Redmond, WA, USA). The dataset consisted of 1806 scans from 107 participants, all acquired under a standardized scanning protocol following predefined SOP guidelines to ensure consistent measurement conditions and minimize operator-dependent variability. Surface scans were scheduled at four time points: (1) before treatment (screening), (2) after the first treatment, (3) after the 12th treatment, and (4) at a follow-up visit one month after completion of the 12-session treatment course. Because the baseline scans were obtained for screening purposes, some participants had data only at the pre-treatment time point. In addition, several participants dropped out during the treatment course, and thus not all participants completed all four measurements. At each available time point, 2–3 repeated scans were collected to ensure reliability, and scans with minimal artifacts were selected for analysis. Each participant underwent repeated 3D surface scanning under two postures at a distance of 750 mm from the camera: a natural upright standing position (P1) and a forward-bending position starting from the upright posture (P2) (Figure 1). At each scheduled time point, 2–3 scans were collected per posture to ensure reliability. Thus, when all four time points were available, each participant contributed approximately 16–24 scans in total (8–12 scans per posture).

**Figure 2:**
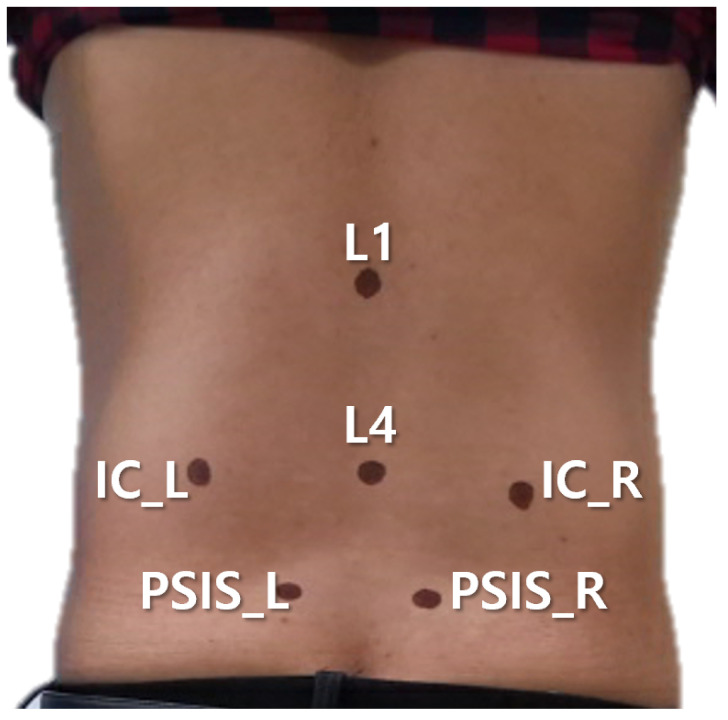
Locations and labels of six anatomical landmarks on the participant’s back.

Each scan was represented as a point cloud that contains approximately 200,000 to 600,000 points, depending on the participant’s body shape, body surface area, and posture (P1 or P2). For the measurements, six anatomical landmarks were first identified by trained clinicians through palpation and then marked on the posterior pelvic surface using a fine tipped marker pen (Figure 2): the bilateral posterior superior iliac spines (PSIS), the bilateral iliac crests (IC), and the vertebral levels L1 and L4. After scanning, a clinical research coordinator (CRC) manually identified the centers of six markers on the reconstructed 3D surface plot via mouse-based digitization, thereby obtaining the 3D coordinates corresponding to each anatomical landmark (PSIS_L, PSIS_R, IC_L, IC_R, L1, and L4).

### 2.2. Overview of the Pipeline

**Figure 3:**
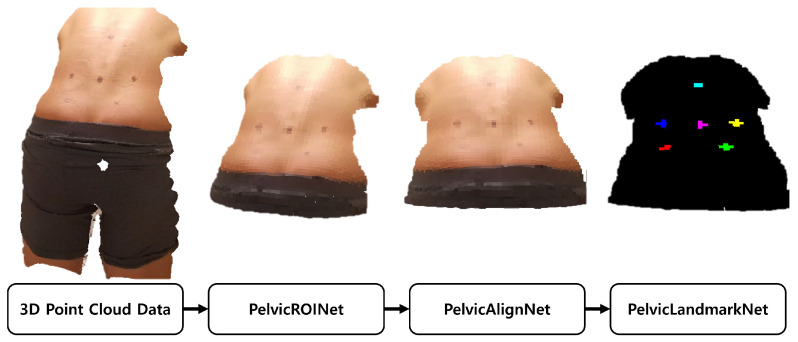
Pipeline of overall procedure.

**Figure 4:**
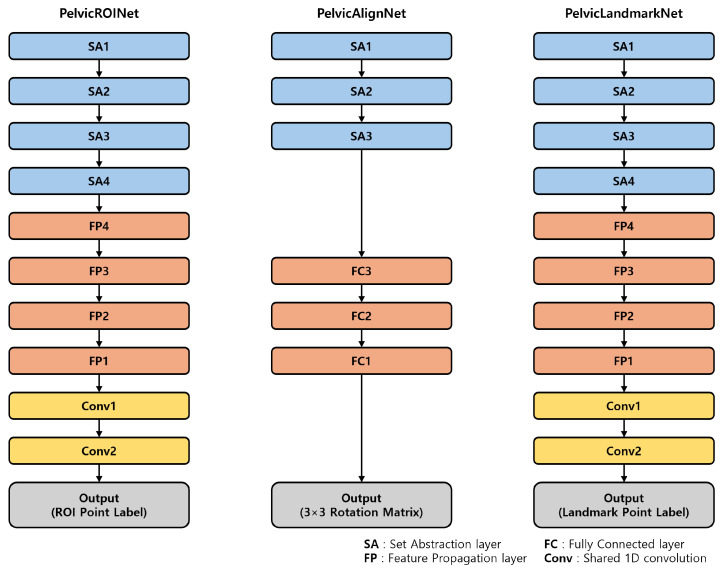
Overview of the three-module pipeline consisting of PelvicROINet, PelvicAlignNet, and PelvicLandmarkNet. Each module adopts a PointNet++ based backbone with task-specific output layers.

Pelvic ROINet extracts the posterior pelvic region of interest (ROI) from the raw 3D point cloud data covering the region from the bilateral PSIS to L1. The extracted ROI is then processed by PelvicAlignNet, which performs rotation correction to align the point cloud to a standardized posture. Finally, PelvicLandmarkNet localizes the coordinates of six anatomical landmarks (bilateral PSIS, bilateral IC, L1, and L4) on the rotation corrected surface data. Each model was trained independently and their outputs were sequentially integrated during inference (Figure 3). The internal architectures of the three modules were based on a PointNet++ backbone, and their structures are summarized in Figure 4.

### 2.3. PelvicROINet

#### 2.3.1. Input & Preprocessing

For each point cloud dataset, the 3D coordinates (x, y, z) of the six markers were used to determine the minimum and maximum values along each axis. All coordinates are expressed in millimeters. Based on these values, the posterior pelvic ROI was defined as

(1) $$[\left(x_{\min}-100,\;x_{\max}+100\right),\left(y_{\min}-100,\;y_{\max}+100\right),\left(z_{\min}-100,\;z_{\max}+100\right)].$$

Points within the ROI were labeled as 1, whereas outside the ROI were labeled as 0. Each point cloud was downsampled to 16,384 points using random uniform sampling without replacement to ensure computational efficiency during training. The dataset was randomly divided into training and test sets at a ratio of 8:2 at the subject level. To prevent data leakage, all scans from a given subject were assigned exclusively to either the training or the test set. Specifically, 20 % of subjects were randomly selected for the test set, and all scans from those subjects were included only in the test set. For data augmentation, each point cloud was translated to its centroid and randomly rotated between minus twenty and plus twenty degrees around the x, y, and z axes, resulting in a twentyfold increase in the number of training samples. In addition, although scans were acquired under a standardized operating procedure (SOP), the dataset naturally included clinically realistic acquisition artifacts, such as partial obstruction of the pelvic region due to incomplete arm abduction, inclusion of surrounding objects, and variations in sampling density depending on the scanner-to-subject distance and viewing angle. This inherent variability complemented the synthetic augmentation and improved robustness under practical scanning conditions. Each point was represented by six input channels, including the raw 3D coordinates and the normalized coordinates relative to the bounding box. Additionally, the set abstraction (SA) layers of PointNet++ [21] also take the raw XYZ coordinates as separate channels for distance computation, the first SA layer receives a total of 9 input channels.

#### 2.3.2. Network Architecture

The model was designed based on the PointNet++ framework for semantic segmentation [20,21,28], consisting of four SA layers followed by four feature propagation (FP) layers. The SA layers progressively downsampled the input point cloud to 1024, 256, 64, and 16 points, respectively, while aggregating local features through multi-scale grouping. The feature dimension was expanded from 9 input channels to 512 channels across these layers. Subsequently, the FP layers hierarchically interpolated and propagated the features back to the original point resolution, producing 128-dimensional point-wise feature representations. Finally, two shared 1D convolutional layers with 128 and C output channels, where C denotes the number of classes, were applied to the propagated features. Each layer was followed by batch normalization, ReLU activation, and dropout (*p* = 0.5). Per-point labeling was obtained using a log-softmax activation.

#### 2.3.3. Loss Function

The negative log-likelihood (NLL) loss was employed to measure the discrepancy between the predicted per-point class probabilities and the ground-truth labels. Optional class weighting was applied to mitigate class imbalance. The NLL loss is defined as

(2) $$L_{\mathrm{NLL}}=-\frac{1}{N}\sum_{i=1}^{N}w_{y_{i}}\log p_{i,y_{i}},$$

where *N* is the number of points, $p_{i,y_{i}}$ is the predicted probability of the *i*-th point for its ground-truth class $y_{i}$, and $w_{y_{i}}$ is the corresponding class weight. For PelvicROINet, the training loss is defined as

(3) $$L_{\mathrm{ROI}}=L_{\mathrm{NLL}}.$$

#### 2.3.4. Training and Evaluation

The model was trained for 20 epochs using the Adam optimizer with a batch size of 32. Evaluation was performed on the held out test set without applying gradient updates. The performance metrics included class-specific Intersection over Union (IoU) and recall for the pelvic ROI.

### 2.4. PelvicAlignNet

#### 2.4.1. Input & Preprocessing

The point clouds extracted by PelvicROINet were used as input for PelvicAlignNet. Each point cloud was translated so that the midpoint of the bilateral PSIS coordinates aligned with the origin, and then rotated around the y axis to horizontally align the PSIS points. Subsequently, the point cloud was rotated around the x axis to set the z coordinate of L4 to zero, thereby establishing a standardized posture. Data augmentation was performed using the same procedure as in PelvicROINet. Each point was represented by six input channels, including the raw 3D coordinates and the normalized coordinates relative to the bounding box. In the set abstraction layers of PointNet++, the raw XYZ coordinates were additionally used for distance computation, resulting in nine input channels in the first SA layer.

#### 2.4.2. Network Architecture

The model is based on the PointNet++ framework and comprises three SA layers. The first two SA layers progressively downsample the point cloud to 512 and 128 points, respectively, while the final SA layer performs global feature aggregation over all input points to produce a global feature vector. This global feature vector is subsequently fed into three fully connected layers to predict a 3 × 3 rotation matrix. Batch-wise singular value decomposition (SVD) is applied to normalize the predicted rotation matrix and enforce its orthonormality.

#### 2.4.3. Loss Function

Training minimizes a weighted sum of three loss terms, including a point-set distance loss between the transformed point cloud and the reference point cloud, a rotation orthonormality regularization loss, and an Euler-angle loss. The point-set distance loss $L_{\mathrm{pts}}$ is defined as the average point-wise Euclidean distance between the predicted transformed points and the reference points.

(4) $$L_{\mathrm{pts}}=\frac{1}{BN}\sum_{b=1}^{B}\sum_{i=1}^{N}{∥{\hat{p}}_{b,i}-p_{b,i}∥}_{2},$$

with ${\hat{p}}_{b,i}$ and $p_{b,i}$ denoting the *i*-th predicted and reference point in the *b*-th batch, respectively. The rotation regularization term $L_{\mathrm{rot}}$ encourages the predicted rotation matrix ${\hat{R}}_{b}$ to be orthonormal.

(5) $$L_{\mathrm{rot}}=\frac{1}{B}\sum_{b=1}^{B}{∥I-{\hat{R}}_{b}^{⊤}{\hat{R}}_{b}∥}_{F},$$

where I is the 3×3 identity matrix and ${∥\cdot ∥}_{F}$ denotes the Frobenius norm. In addition, the Euler-angle loss is computed as the $\ell_{2}$ distance between Euler angles converted from the predicted and reference rotation matrices.

(6) $$L_{\mathrm{Euler}}=\frac{1}{B}\sum_{b=1}^{B}{∥\theta \left({\hat{R}}_{b}\right)-\theta \left(R_{b}\right)∥}_{2},$$

where θ(·) denotes the Euler-angle representation $\left(\theta_{x},\theta_{y},\theta_{z}\right)$ derived from a rotation matrix. The weighting factors $\lambda_{\mathrm{rot}}$ and $\lambda_{\mathrm{Euler}}$ control the contributions of the rotation regularization and Euler-angle terms, respectively. The total loss of PelvicAlignNet is defined as

(7) $$L_{\mathrm{Align}}=L_{\mathrm{pts}}+\lambda_{\mathrm{rot}}L_{\mathrm{rot}}+\lambda_{\mathrm{Euler}}L_{\mathrm{Euler}}.$$

#### 2.4.4. Training and Evaluation

The model was trained using the Adam optimizer with a batch size of 32. Evaluation on the held-out test set included the mean distance between the predicted and reference point clouds, as well as the rotation regularization loss.

### 2.5. PelvicLandmarkNet

#### 2.5.1. Input & Preprocessing

The input to PelvicLandmarkNet consisted of the rotation corrected pelvic surface point clouds obtained from PelvicROINet and PelvicAlignNet. To address the imbalance between background and marker points, all points within a 10 mm radius around each anatomical marker were assigned the same label during training, the average coordinates of the predicted regions were then compared with the ground-truth marker coordinates. For each of the six anatomical markers, points within a 10 mm radius from the marker center were labeled as 1–6, respectively, while all other points were labeled as 0. Data augmentation followed the same procedure as in PelvicROINet. Each point was represented by six feature channels, including the raw 3D coordinates and normalized coordinates relative to the bounding box. Additionally, the SA layers of PointNet++ use the raw XYZ coordinates as separate input channels, resulting in a total of nine input channels for the first SA layer.

#### 2.5.2. Network Architecture

The network architecture of PelvicLandmarkNet is similar to PelvicROINet. Both networks use the same PointNet++ backbone and share the same overall structure.

#### 2.5.3. Loss Function

The total loss used for training the PelvicLandmarkNet model consists of two terms, a negative log-likelihood loss for point-wise label prediction and a distance-based loss that penalizes deviations between the predicted and ground-truth marker centers. The negative log-likelihood loss follows the same formulation as defined in Equation (2).

The distance-based loss is designed to encourage accurate localization of marker centers. It is computed as the average Euclidean distance between the predicted and ground-truth marker centers over all valid markers and batches.

(8) $$L_{\mathrm{dist}}=\frac{1}{B}\sum_{b=1}^{B}\frac{1}{K_{b}}\sum_{k=1}^{K_{b}}{∥{\hat{m}}_{b,k}-m_{b,k}∥}_{2},$$

where $K_{b}$ denotes the number of surface markers present in the ground truth for the *b*-th batch, and ${\hat{m}}_{b,k}$ and $m_{b,k}$ represent the predicted and ground-truth centers of the *k*-th marker, respectively. The overall objective is given by

(9) $$L_{\mathrm{Landmark}}=L_{\mathrm{NLL}}+\lambda_{\mathrm{dist}}\;L_{\mathrm{dist}},$$

where the weighting factor $\lambda_{\mathrm{dist}}$ controls the contribution of the distance-based term.

#### 2.5.4. Training and Evaluation

The model was trained for 20 epochs with the Adam optimizer and a batch size of 32. Evaluation on the held-out test set was performed using the mean Euclidean distance between predicted and ground-truth landmark coordinates and the overall point-wise labeling accuracy.

## 3. Results

**Figure 5:**
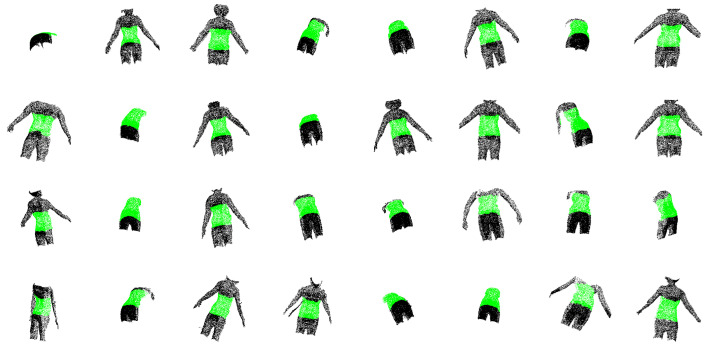
Examples of segmentation results of PelvicROINet.

**Figure 6:**
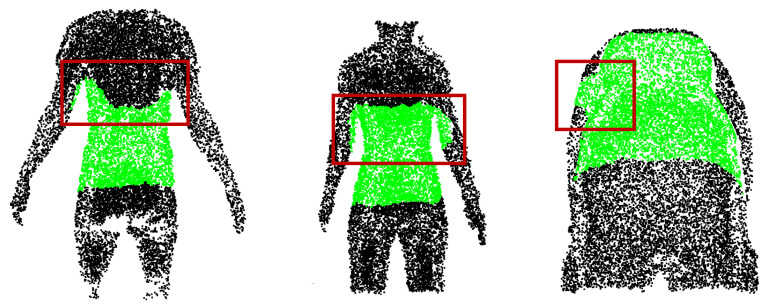
Representative failure cases of pelvic ROI extraction. Red boxes highlight regions affected by clothing occlusion, over-segmentation toward the shoulder region, and unintended inclusion of the upper limb.

The quantitative and qualitative performance of each module and the fully integrated pipeline are summarized below. For ROI extraction, class-specific metrics were analyzed to avoid bias from class imbalance. On the evaluation dataset, PelvicROINet achieved an IoU of 0.908 and a recall of 0.957 for the pelvic surface region, while the corresponding IoU and recall for the others region were 0.955 and 0.975, respectively. These results indicate that the model reliably captures the pelvic ROI while maintaining high segmentation performance across both classes. Figure 5 shows qualitative examples of segmentation results obtained by PelvicROINet. The green regions correspond to the pelvic surface, whereas the black regions indicate the other region. To further illustrate model behavior under challenging conditions, representative failure cases are shown in Figure 6. These examples include partial occlusion by clothing, over-segmentation toward adjacent upper-body regions, and unintended inclusion of portions of the upper limb within the predicted ROI.

**Figure 7:**
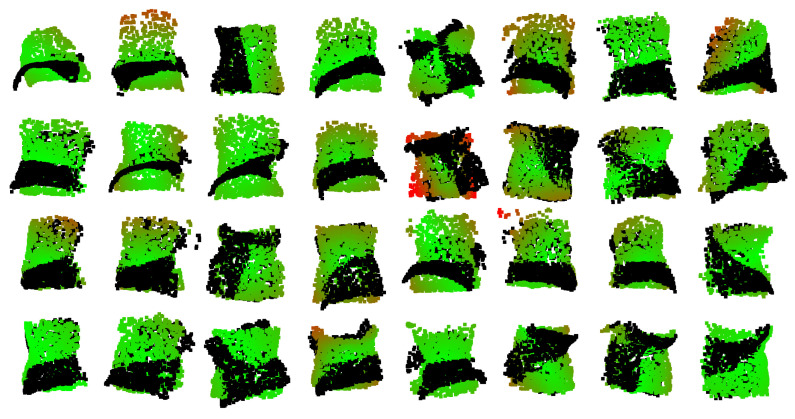
Examples of alignment results obtained by PelvicAlignNet.

PelvicAlignNet effectively learned the rigid alignment between augmented and original point clouds. During training, the model minimized both the Euler loss (radian) and the mean normalized point-to-point Euclidean distance (millimeter), achieving 0.0228 rad and 2.19 mm, respectively. In the test phase, the corresponding losses were 0.0369 rad and 4.64 mm, confirming that the model maintained stable performance on unseen data. The small differences between training and test losses suggest that PelvicAlignNet generalized well without noticeable overfitting. Figure 7 visualizes representative alignment results. The black points indicate the original input point cloud before rotation correction, while the rotation-corrected points are overlaid and color-coded according to their distances from the ground-truth reference, ranging from green (0.0 mm) to red (10.0 mm), with greener areas indicating smaller deviations from the reference alignment.

**Figure 8:**
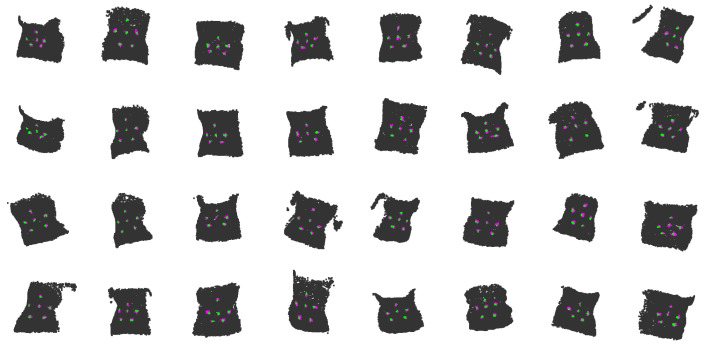
Examples of landmark localization results obtained by PelvicLandmarkNet.

PelvicLandmarkNet achieved precise localization performance under the subject-level split setting. The average Euclidean distance between the predicted and ground-truth landmark coordinates was 5.99 mm in training and 11.97 mm in testing when $\lambda_{\mathrm{dist}}$ = 10, while the point-wise labeling accuracy reached 98.05 % and 97.05 %, respectively, reflecting accurate assignment of surface points to their corresponding landmark regions. These results confirm that PelvicLandmarkNet effectively localized the anatomical landmarks with high accuracy under a subject-level split, where inter-subject morphological variability presents a more challenging generalization scenario. Figure 8 presents qualitative examples of landmark localization results: gray points represent the pelvic surface after augmentation, magenta points indicate the predicted landmark positions, and green points mark where the predicted and ground-truth landmarks overlap.

To quantitatively evaluate the effect of each processing module on anatomical landmark localization, five model configurations were tested and compared. The evaluated configurations included: (1) the baseline model, which directly applied PointNet++ to the raw body-surface point cloud; (2) manually cropped configuration (Manual Crop) followed by PelvicLandmarkNet; (3) manually cropped region with PelvicAlignNet and PelvicLandmarkNet; (4) PelvicROINet followed by PelvicLandmarkNet; and (5) the fully integrated pipeline combining PelvicROINet, PelvicAlignNet, and PelvicLandmarkNet. The dataset was split at the subject level to avoid subject leakage between training and testing. The test set consisted of 22 independent subjects, yielding 396 point-cloud acquisitions and 2376 landmark instances (6 landmarks per scan). The baseline model directly localized landmarks using PointNet++ on the body-surface point clouds without PelvicROINet or PelvicAlignNet, with uniform random downsampling applied for computational efficiency. For the Manual Crop configurations, the region of interest was defined using the same bounding formulation as in Equation (1), with the fixed 100 mm margin replaced by randomly sampled offsets ranging from 50 to 150 mm for each lower and upper bound along all three axes. Independent random offsets within ±50 mm of 100 mm were applied to the minimum and maximum coordinates for each axis on a per-sample basis to approximate the variability inherent in clinician-guided visual ROI selection. The resulting point clouds were then used as input for the landmark localization and alignment modules.

**Figure 9:**
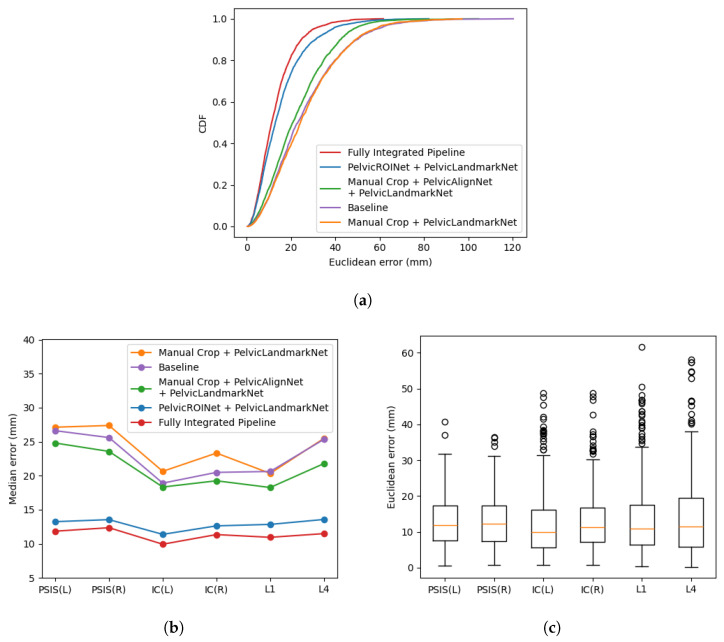
Distribution analysis of landmark localization performance. (**a**) CDF of Euclidean distance error for all models. (**b**) Median error comparison. (**c**) Boxplot of localization error for the fully integrated pipeline.

Figure 9 presents the landmark localization performance across different model configurations. As shown in Figure 9a, the cumulative distribution functions (CDFs) indicate that the fully integrated pipeline dominated the other configurations across most distance thresholds, exhibiting a steeper cumulative rise and consistently lower localization errors. In contrast, the baseline model exhibited the broadest error distribution. The progressive contribution of each module is further reflected in Figure 9b, where the median localization error decreases as preprocessing components are incrementally introduced. The fully integrated pipeline achieved the lowest overall median error of 11.25 mm. The boxplot in Figure 9c illustrates the variability of localization errors for the fully integrated pipeline. Although several outliers were observed, the interquartile range remained compact, with per-landmark median errors ranging from 10.02 to 12.12 mm, indicating stable performance across most cases. At the subject level (n = 22), the fully integrated pipeline yielded a mean localization error of 13.22 mm (95% CI: 11.76–14.68 mm), consistent with the overall median-based observations. In addition to localization performance, computational efficiency was evaluated for each configuration. As summarized in Table 1, the baseline model exhibited the fastest processing time (0.73 ± 0.10 s/sample), whereas the fully integrated pipeline required 3.11 ± 0.43 s/sample due to the additional preprocessing modules. Despite the increased computational cost, the integrated framework achieved substantially improved localization accuracy, demonstrating a favorable trade-off between precision and processing time.

Following the comparison of localization performance, the repeatability of landmark localization was further examined to validate the reliability of the proposed model under repeated measurement conditions. Two or three repeated surface scans acquired within the same visit were analyzed. Within-visit repeatability was evaluated by calculating the standard deviation (STD) and coefficient of variation (CV) of the distances between paired anatomical landmarks (PSIS_L–PSIS_R, IC_L–IC_R, and L1–L4). In addition, the intraclass correlation coefficient (ICC) was computed to assess measurement consistency across repeated scans. The same analyses were applied to the landmarks manually annotated by clinical experts to serve as a reference. The results are summarized in Table 2. Overall, the fully integrated pipeline demonstrated smaller STD and CV values and higher ICC values than manual landmark digitization, indicating improved repeatability and more consistent measurements. The improvement was most evident in the PSIS region, where the CV decreased from 6.39% (manual) to 4.20%, and the ICC increased from 0.62 to 0.91, suggesting that the fully integrated pipeline effectively reduced variability arising from user dependent factors during repeated data acquisition.

Finally, to strengthen methodological robustness and support parameter sensitivity validation, we conducted additional sensitivity analyses on key hyperparameters affecting the final landmark localization performance. The results are summarized in Table 3. For the loss formulation in Equation (9), we evaluated $\lambda_{\mathrm{dist}}\in \left\{5,10,15\right\}$ while fixing the ROI margin to 10 mm. The results indicate that $\lambda_{\mathrm{dist}}=10$ achieved the lowest localization error on the test set. However, performance variations across the tested values were minimal, suggesting that the proposed framework is not highly sensitive to moderate changes in the loss weight. In addition, the sensitivity of the landmark labeling radius was evaluated using radii of 10, 15, and 20 mm while fixing $\lambda_{\mathrm{dist}}$ = 10. During label generation, points within the specified radius from each ground-truth marker coordinate were assigned to the corresponding landmark class. The lowest mean Euclidean error was observed at a radius of 15 mm. However, performance differences across 10–15 mm remained within approximately 0.5 mm on the test set. Given this minimal variation and to maintain methodological consistency with the training configuration, a radius of 10 mm was adopted for the final model.

## 4. Discussion

This study demonstrates that a modular, hierarchical pipeline enables reliable anatomical landmark localization on 3D human surface data. By sequentially applying region of interest (ROI) extraction, pose alignment, and landmark localization, the proposed system achieved low localization error under a subject-disjoint evaluation protocol. Notably, the fully integrated pipeline dominated alternative configurations across most distance thresholds in the cumulative distribution analysis and exhibited a stepwise reduction in median error as preprocessing modules were incrementally introduced. These findings indicate that hierarchical preprocessing substantially enhances localization stability compared with single-stage regression directly applied to raw point clouds.

The quantitative comparison across five model configurations provides clear evidence of progressive module contribution. The baseline PointNet++ model showed broader error distributions and higher median localization error, suggesting sensitivity to posture variation, irrelevant surface regions, and anatomical ambiguity. Introducing a manually defined ROI yielded only marginal improvement, indicating that non-learned cropping is insufficient to ensure input consistency. In contrast, replacing manual cropping with PelvicROINet significantly reduced irrelevant spatial information, and the addition of PelvicAlignNet further stabilized geometric orientation. The fully integrated pipeline achieved the lowest overall localization error, confirming that learned ROI restriction and explicit geometric alignment act synergistically to improve downstream landmark localization. A closer inspection of the segmentation results further reveals characteristic failure patterns that provide insight into the model’s practical robustness, despite the high overall segmentation performance of PelvicROINet. As illustrated in Figure 6, partial occlusion by clothing occasionally reduced the visible surface, leading to incomplete ROI coverage. In addition, over-segmentation toward the shoulder region was observed in some cases, particularly when upper-body posture deviated from the standardized operating procedure. Furthermore, partial inclusion of the upper limb occurred when arm abduction was insufficient, resulting in spatial proximity between the arm and pelvic boundary. Nevertheless, although occasional mis-segmentation artifacts were observed, the predicted ROI generally provided sufficient coverage of the six target landmark regions. Consequently, the performance of the downstream alignment and landmark localization modules was not substantially degraded, suggesting a degree of robustness to moderate ROI imperfections. The structural roles of the individual modules explain this collective effectiveness. The ROI extraction step restricts analysis to anatomically relevant posterior pelvic regions, reducing noise from surrounding body surfaces. The alignment module minimizes pose-induced variability, enabling the subsequent landmark localization module to learn orientation-invariant geometric features rather than posture-dependent cues. This hierarchical design closely mirrors the clinical workflow in which clinicians first define the region of interest, standardize the patient’s posture, and then identify anatomical landmarks. Such alignment between computational and clinical workflows enhances the interpretability and applicability of the proposed system.

Under the subject-level split setting, the fully integrated pipeline achieved a mean landmark localization error of 13.22 mm (95% CI: 11.76–14.68 mm), with a median error of 11.25 mm. Kilby et al. reported mean localization errors of approximately 15–20 mm for lumbar and pelvic landmarks, with limits of agreement approaching ±27 mm relative to ultrasound reference standards [29]. More recently, Hvidkær et al. observed a mean palpation precision of approximately 13 mm (95% CI: 11–15 mm) using 3D surface-based assessment [30]. The error magnitude and confidence interval observed in the present study closely overlap with the range reported for experienced clinicians and remain below the mean errors reported in earlier ultrasound-referenced investigations. These findings suggest that the proposed fully integrated pipeline achieves a level of landmark localization precision comparable to skilled manual palpation in routine clinical settings, while providing consistent and reproducible measurements across subjects.

The repeatability analysis further supports this interpretation. Across repeated within-visit scans, the integrated pipeline demonstrated lower standard deviation and coefficient of variation values compared with manual landmark marking, particularly in the PSIS region where the CV decreased from 6.39% to 4.20%. Intraclass correlation coefficients ranged from 0.78 to 0.91 for the automated pipeline, clearly exceeding the reliability range observed for manual measurements. These results indicate that automated landmark estimation reduces operator-dependent variability inherent in manual digitization procedures and provides greater consistency under repeated acquisition conditions, which may reduce the impact of posture-related soft tissue displacement on repeated measurements [6,25]. Improved repeatability strengthens the suitability of the proposed system for longitudinal monitoring, post-treatment follow-up evaluation, and routine musculoskeletal assessment.

To assess methodological robustness, sensitivity analyses were conducted for key hyperparameters affecting landmark localization. Variations in the loss weight and labeling radius resulted in changes of less than approximately 0.5 mm in test error, indicating stable performance across reasonable parameter ranges. These findings suggest that performance gains primarily stem from the hierarchical pipeline design rather than narrow hyperparameter tuning.

PointNet++ was selected as a validated and reproducible baseline architecture for geometric feature learning from raw point clouds. The primary contribution of this study lies not in proposing a novel backbone network, but in the modular integration of learned ROI restriction, geometric alignment, and landmark regression within a clinically structured workflow. While more advanced architectures may provide additional modeling capacity, the present study emphasizes workflow-oriented design and surface-geometry-driven consistency.

From a computational perspective, the entire inference process required approximately 3.11 s per sample on a workstation equipped with an AMD Ryzen 7 5800X CPU and an RTX 4090 GPU. In a practical clinical workflow, near-immediate landmark output supports a clinician-in-the-loop process in which predicted landmarks can be rapidly reviewed and corrected if necessary, potentially reducing operator workload and fatigue-related variability.

Several limitations should be acknowledged. The dataset used for training mainly included upright and forward bending postures, which may limit generalization to more diverse body configurations. Landmark annotations were based on clinician judgment [31,32] and may incorporate subtle soft tissue displacement effects [33,34], which could influence the learning process. In addition, the present model focuses on six posterior pelvic landmarks, and extension to other anatomical regions requires further validation.

Despite these limitations, the proposed modular framework demonstrated robust performance in a region characterized by relatively subtle anatomical surface cues. The modular architecture allows each component to be independently retrained or replaced in future studies, enabling extension to other anatomical regions or alternative module configurations for broader clinical and biomechanical applications. Future work may include incorporation of larger and more diverse datasets, exploration of advanced geometric learning architectures, optimization for lightweight deployment environments, and multimodal fusion strategies combining point cloud data with complementary imaging modalities. With continued validation and methodological refinement, the proposed framework may contribute to more objective and repeatable surface-based assessments for musculoskeletal evaluation and longitudinal monitoring.

## 5. Conclusions

This study developed a modular deep learning pipeline for automatic localization of anatomical landmarks from 3D pelvic surface point clouds. By integrating PelvicROINet, PelvicAlignNet, and PelvicLandmarkNet, the system enabled fully automated analysis without manual intervention, improving both accuracy and reproducibility. The fully integrated pipeline achieved reliable landmark localization under a subject-level split evaluation setting, with a median error of 11.25 mm. These results confirm the feasibility of near real time clinical application and highlight the potential of the proposed modular framework as a foundation for future AI-assisted musculoskeletal assessment and diagnosis.

## Figures and Tables

**Table 1:** Average processing time per point cloud sample for each model configuration.

Model Combination	Time (s/Sample)
Baseline	0.73 ± 0.10
Manual Crop + PelvicLandmarkNet	0.76 ± 0.10
Manual Crop + PelvicAlignNet + PelvicLandmarkNet	1.38 ± 0.17
PelvicROINet + PelvicLandmarkNet	2.49 ± 0.43
**Fully Integrated Pipeline**	3.11 ± 0.43

**Table 2:** Repeatability comparison between the integrated pipeline (ROI + Align + LM) and manual landmark marking.

Measurement Region	Fully Integrated Pipeline			Manual Marking		
	STD (mm)	CV (%)	ICC (95% CI)	STD (mm)	CV (%)	ICC (95% CI)
PSIS_L–PSIS_R	3.86	4.20	0.91 (0.88–0.93)	6.35	6.39	0.62 (0.53–0.70)
IC_L–IC_R	2.34	1.57	0.78 (0.72–0.83)	3.22	2.18	0.52 (0.42–0.62)
L1–L4	4.29	5.32	0.87 (0.82–0.90)	5.94	7.22	0.77 (0.71–0.83)

**Table 3:** Sensitivity analysis of key hyperparameters affecting landmark localization performance (test set).

Parameter	Value	Mean Euclidean Error (mm)
Loss weight $\lambda_{\mathrm{dist}}$ (Labeling radius = 10 mm)	5	12.29
	10	11.97
	15	12.19
Labeling radius (mm) ($\lambda_{\mathrm{dist}}=10$)	10	11.97
	15	11.49
	20	11.66

## Data Availability

The data presented in this study are available upon reasonable request.

## References

[1] D’Amico M., Kinel E., D’Amico G., Roncoletta P. (2021). A Self-Contained 3D Biomechanical Analysis Lab for Complete Automatic Spine and Full Skeleton Assessment of Posture, Gait and Run. Sensors.

[2] Balasubramanian M., Sheykhmaleki P. (2025). Emerging Roles of 3D Body Scanning in Human-Centric Applications. Technologies.

[3] Cruz-Medel I., Rodrigues-de Souza D.P., Alburquerque-Sendín F. (2024). Comprehensive Analysis of Pelvic Asymmetries in Low Back Pain, Scoliosis, Post-Traumatic Pelvic Dysfunctions and Obstetric Changes: A Narrative Review Focused on Clinical Relevance. Symmetry.

[4] Bibrowicz K., Szurmik T., Ogrodzka-Ciechanowicz K., Hudakova Z., Gąsienica-Walczak B., Kurzeja P. (2023). Asymmetry of the pelvis in Polish young adults. Front. Psychol..

[5] Rayward L., Pearcy M., Izatt M., Green D., Labrom R., Askin G., Little J.P. (2023). Predicting spinal column profile from surface topography via 3D non-contact surface scanning. PLoS ONE.

[6] Wolf C., Betz U., Huthwelker J., Konradi J., Westphal R.S., Cerpa M., Lenke L., Drees P. (2021). Evaluation of 3D vertebral and pelvic position by surface topography in asymptomatic females: Presentation of normative reference data. J. Orthop. Surg. Res..

[7] Kang T.H., Jang S., Seo I., Choi M., Park Y., Lee Y., Lee J.H., Cho M. (2025). A new 3D full-body scanner analyzing the sagittal and coronal balance of the adult spine: A preliminary prospective observational study. Acta Neurochir..

[8] Harrison D.E., Janik T.J., Cailliet R., Harrison D.D., Normand M.C., Perron D.L., Oakley P.A. (2008). Upright Static Pelvic Posture as Rotations and Translations in 3-Dimensional From Three 2-Dimensional Digital Images: Validation of a Computerized Analysis. J. Manip. Physiol. Ther..

[9] González-Ortega D., Díaz-Pernas F.J., Martínez-Zarzuela M., Antón-Rodríguez M. (2014). A Kinect-based system for cognitive rehabilitation exercises monitoring. Comput. Methods Programs Biomed..

[10] Haleem A., Javaid M. (2019). 3D scanning applications in medical field: A literature-based review. Clin. Epidemiol. Glob. Health.

[11] Zurawski A.L., Friebe D., Zaleska S., Wojtas K., Gawlik M., Wilczyński J. (2025). Assessing Trunk Cross-Section Geometry and Spinal Postures with Noninvasive 3D Surface Topography: A Study of 108 Healthy Young Adults. Sensors.

[12] Sun Y., Zhang X., Miao Y. (2024). A review of point cloud segmentation for understanding 3D indoor scenes. Vis. Intell..

[13] Xu T., An D., Jia Y., Yue Y. (2021). A Review: Point Cloud-Based 3D Human Joints Estimation. Sensors.

[14] Zhou L., Sun G., Li Y., Li W., Su Z. (2022). Point cloud denoising review: From classical to deep learning-based approaches. Graph. Models.

[15] Han X.F., Jin J.S., Wang M.J., Jiang W., Gao L., Xiao L. (2017). A review of algorithms for filtering the 3D point cloud. Signal Process. Image Commun..

[16] Besl P.J., McKay N.D. (1992). A Method for registration of 3-D shapes. IEEE Trans. Pattern Anal. Mach. Intell..

[17] Zhang J., Yao Y., Deng B. (2022). Fast and Robust Iterative Closest Point. IEEE Trans. Pattern Anal. Mach. Intell..

[18] Cooperstein R., Hickey M. (2016). The reliability of palpating the posterior superior iliac spine: A systematic review. J. Can. Chiropr. Assoc..

[19] Al-baker B., Alkalaly A., Ayoub A., Ju X., Mossey P. (2023). Accuracy and reliability of automated three-dimensional facial landmarking in medical and biological studies. A systematic review. Eur. J. Orthod..

[20] Qi C.R., Su H., Mo K., Guibas L.J. (2017). PointNet: Deep Learning on Point Sets for 3D Classification and Segmentation. arXiv.

[21] Qi C.R., Yi L., Su H., Guibas L.J. (2017). PointNet++: Deep Hierarchical Feature Learning on Point Sets in a Metric Space. arXiv.

[22] Zhao H., Jiang L., Fu C.W., Jia J. PointWeb: Enhancing Local Neighborhood Features for Point Cloud Processing. Proceedings of the IEEE/CVF Conference on Computer Vision and Pattern Recognition (CVPR).

[23] Wang Y., Sun Y., Liu Z., Sarma S.E., Bronstein M.M., Solomon J.M. (2019). Dynamic Graph CNN for Learning on Point Clouds. ACM Trans. Graph..

[24] Li Y., Bu R., Sun M., Wu W., Di X., Chen B. (2018). PointCNN: Convolution On X-Transformed Points. Proceedings of the Advances in Neural Information Processing Systems.

[25] Camomilla V., Bonci T., Cappozzo A. (2017). Soft Tissue Displacement over Pelvic Anatomical Landmarks during 3-D Hip Movements. J. Biomech..

[26] Abreu de Souza M., Alka Cordeiro D.C., Oliveira J.d., Oliveira M.F.A.d., Bonafini B.L. (2023). 3D Multi-Modality Medical Imaging: Combining Anatomical and Infrared Thermal Images for 3D Reconstruction. Sensors.

[27] Zhang T., Liang Z., Wang B. (2025). A Survey of Medical Point Cloud Shape Learning: Registration, Reconstruction and Variation. arXiv.

[28] Yan X. Pointnet/Pointnet++ Pytorch, 2019. https://github.com/yanx27/Pointnet_Pointnet2_pytorch.

[29] Kilby J., Heneghan N.R., Maybury M. (2012). Manual palpation of lumbo-pelvic landmarks: A validity study. Man. Ther..

[30] Hvidkær I.S., Harsted S., Hadizadeh M., O’Neill S., Kawchuk G.N., Nim C. (2024). Static palpation ain’t easy: Evaluating palpation precision using a topographical map of the lumbar spine as a reference. PLoS ONE.

[31] Della Croce U., Cappozzo A., Kerrigan D.C. (1999). Pelvis and lower limb anatomical landmark calibration precision and its propagation to bone geometry and joint angles. Med. Biol. Eng. Comput..

[32] Fagertun J., Harder S., Rosengren A., Moeller C., Werge T., Paulsen R.R., Hansen T.F. (2014). 3D facial landmarks: Inter-operator variability of manual annotation. BMC Med. Imaging.

[33] Lavaill M., Martelli S., Kerr G.K., Pivonka P. (2022). Statistical Quantification of the Effects of Marker Misplacement and Soft-Tissue Artifact on Shoulder Kinematics and Kinetics. Life.

[34] Ryu T. (2012). Application of Soft Tissue Artifact Compensation Using Displacement Dependency between Anatomical Landmarks and Skin Markers. Anat. Res. Int..

